# The Influence of Mind

**Published:** 1896-04

**Authors:** C. H. Hughes


### The Influence of Mind.

Great brain and nerve strain, as in insanity, brittles the bones; grief and fright blanches the face and hair; fear paralyzes the heart, depresses the temperature, causes excessive and clammy perspiration; anxiety arrests secretion and shrivels the skin; remorse wastes away the body; anger flushes the face and so fills the brain with blood that its vessels burst and the victims fall with apoplexy; shame flushes the cheek, slows the heart and respiration; sorrow shows itself in tears; love and good fortune brighten the countenance and quicken the step and pulse and lift up the form; while adversity and remorse sadden the face, slow the pulse, bend the form, and depress the bodily movements. These things, and many needless to mention, show us the potency of mental influence, through its proper neural channels, on the movements of the organism. We cannot deny them in regard to the stomach. On the contrary, as we see the systole of the heart arrested by emotion, so we see digestion stayed by disagreeable and depressing thought. Mental force, through psycho-neural media, pervades the body, and the stomach is not exempt from its invigorating or depressing influence over its physiologic functions.—C. H. Hughes, M.D.

